# Editorial: Wearable and Implantable Technologies in the Rehabilitation of Patients With Sensory Impairments

**DOI:** 10.3389/fnins.2021.740263

**Published:** 2021-08-11

**Authors:** Cristian F. Pasluosta, Milos. R. Popovic, Bjoern M. Eskofier, Thomas Stieglitz

**Affiliations:** ^1^Laboratory for Biomedical Microtechnology, Department of Microsystems Engineering, University of Freiburg, Freiburg, Germany; ^2^KITE - Research Institute, University Health Network, Toronto, ON, Canada; ^3^Institute of Biomedical Engineering, University of Toronto, Toronto, ON, Canada; ^4^Machine Learning and Data Analytics Lab, Department of Artificial Intelligence in Biomedical Engineering, Friedrich-Alexander-Universität Erlangen-Nürnberg (FAU), Erlangen, Germany; ^5^Bernstein Center Freiburg, University of Freiburg, Freiburg, Germany; ^6^BrainLinks-BrainTools, University of Freiburg, Freiburg, Germany

**Keywords:** sensory feedback, wearable, implants, rehabilitation, biomedical

Sensory feedback is embedded in every physiological control system. Diseases affecting sensory feedback alter these control systems and elicit compensatory mechanisms. Neurological disorders such as Parkinson's disease, stroke, and diabetic neuropathy, as well as limb amputations and loss of hearing are examples of pathologies resulting in sensory impairments. New wearable and implantable technologies are growingly integrated into the rehabilitation of patients with impaired sensory feedback. This Research Topic compiles evidence of how these emerging technologies shape the present and future of disease assessment, monitoring and treatment.

The loss of a limb disrupts sensory information that is essential to maintain a stable upright standing, leading to compensatory adaptations of the intact limb to cope with an impaired postural control system (Claret et al., 2019). In this Research Topic, Shell et al. provided tactile sensory feedback of the foot-sole by electrically stimulating the remaining nerves of transtibial amputees through implanted cuff electrodes. They measured changes in the center of pressure (CoP) position during quiet standing induced by plantar sensations evoked by the neural stimulation. Changes in CoP position were compared with the ones induced by placing vibrating motors under the intact foot. Notably, electrical stimulation of the peripheral pathways and vibration yielded comparable shifts in the CoP position. This indicates that the postural control system incorporates artificial sensory feedback, yet without measurable discrimination of electrical stimulation vs. vibrating the skin of the intact foot-sole.

In another work, Dong et al. compared the use of surface and subdermal electrotactile stimulation to deliver sensory feedback in upper limb amputees. They presented a closed-loop feedback control system using surface or subdermal electrotactile feedback to convey tactile perceptions during a tracking trajectory task. Sensory feedback was delivered as the momentary tracking error. Their findings suggest that even though subdermal is a viable method for sensory feedback, surface electrotactile feedback outperforms it and presents as a more reliable option.

Loss or impaired sensory feedback is also present in stroke survivors. With a novel wearable neurorobotic system, Ferrari et al. delivered 90 Hz vibrotactile feedback to induce illusory kinaesthetic sensations and to reproduce proprioceptive feedback in the upper limb of stroke patients. They observed that illusory sensations by vibrating agonist muscles during reaching tasks produced smoother motion patterns with increased movement directness and increased elbow extension. However, stimulating antagonist muscles resulted in a negative impact on the performance of the same task.

The vagus nerve is an important pathway that drives many cardiovascular variables such us blood pressure. Electrically stimulating the vagus nerve has proven successful to treat epileptic seizures and chronic depression (Nemeroff et al., 2006; Milby et al., 2008; Howland, 2014). In this Research Topic, Yaghouby et al. presented work on electrical stimulation of this pathway in female and male rats to investigate sex differences with respect to response of the cardiovascular and immune systems. They observed significant transient sex differences in cardiovascular activity but no differences on the immune system.

The cochlear implant is one of the most successful implantable devices to restore sensory feedback. Despite the remarkable advancements in the recuperation of hearing, the quality of hearing is far from natural. Fletcher and Verschuur review the literature to explore whether a combination of wearable and implant technologies may improve the outcome of cochlear implants. They remark the necessity of understanding the neural basis of combining these two technologies, and call for optimizing signal processing to enhance the quality of hearing. In another work on audition, Duret et al. investigated musical perception in patients with binaural cochlear implants in relationship to quality of life. They report that bimodal stimulation outperforms unimodal hearing in improving musical perception, and that musical perception seems to be related to quality of life in this patient population.

Assessing treatment outcomes requires objective metrics to measure the impact of new technologies in patient's quality of life. The newly established Dresden protocol for multidimensional walking assessment (DMWA) reported by Trentzsch et al. is a collection of techniques and procedures to evaluate gait in patients with multiple sclerosis. A combination of self-reported assessment, video-based analysis, instrumented walkways and wearable sensors are proposed to record changes in gait patterns from a multi-perspective point of view.

Taken together, the articles compiled in this Research Topic highlight the usefulness of new wearable and implantable technologies to treat patients with various types of loss of sensory feedback. These works also feature the importance of investigating the pathophysiology underlying sensory impairment to better interface technology with the biology of physiological processes.

## Author Contributions

All authors listed have made a substantial, direct and intellectual contribution to the work, and approved it for publication.

## Conflict of Interest

BE reports grants from Portabiles HealthCare Technologies GmbH, grants from adidas AG, outside the work; In addition, BE has a patent related to gait assessments pending. TS is co-founder and member of the scientific advisory board of the spin-off companies CorTec GmbH and neuroloop GmbH. MP is the founder and is on the board of directors of Myndtec; he also holds shares in the company. The remaining author declares that the research was conducted in the absence of any commercial or financial relationships that could be construed as a potential conflict of interest.

## Publisher's Note

All claims expressed in this article are solely those of the authors and do not necessarily represent those of their affiliated organizations, or those of the publisher, the editors and the reviewers. Any product that may be evaluated in this article, or claim that may be made by its manufacturer, is not guaranteed or endorsed by the publisher.
